# Supplementary material for: Bold Frogs or Shy Toads? How Did the COVID-19 Closure of Zoological Organisations Affect Amphibian Activity?
Source: Animals (Basel). 2021 Jul 2;11(7):1982. doi: 10.3390/ani11071982 (PMC8300174; doi:10.3390/ani11071982)
Supplement: Supplementary file 1 [file animals-11-01982-s001.zip › animals-1275686-supplementary.pdf]

Supplementary Table S1: Median temperature (± SD) for each enclosure during the study period.

**Enclosure**	**Month**	**Median temperature °C**
Native	March	18.35 ±0.652
	April	19.1 ±1.118
	May	20.05 ±1.146
	June	22.4 ±1.772
	August	23.65 ±1.722
	September	21.8 ±1.231
Pool frog	March	19.45 ±0.582
	April	19.9 ±1.316
	May	21.2 ±1.075
	June	23.9 ±1.963
	August	24.95 ±1.683
	September	22.3 ±1.201
Mantella	March	19.65 ±1.518
	April	19.9 ±0.958
	May	20.7 ±1.179
	June	23.7 ±1.491
	August	24.65 ±1.653
	September	23.2 ±1.113
Dart frog	March	20.35 ±0.864
	April	22 ±0.744
	May	22.7 ±1.085
	June	25.2 ±1.134
	August	25.2 ±1.126
	September	24.4 ±0.728
